# CD8+CD103+ iTregs Inhibit Chronic Graft-versus-Host Disease with Lupus Nephritis by the Increased Expression of CD39

**DOI:** 10.1016/j.ymthe.2019.07.014

**Published:** 2019-07-26

**Authors:** Xiao Zhang, Xia Ouyang, Zhenjian Xu, Junzhe Chen, Qiuyan Huang, Ya Liu, Tongtong Xu, Julie Wang, Nancy Olsen, Anping Xu, Song Guo Zheng

**Affiliations:** 1 Department of Nephrology, Sun Yat-sen Memorial Hospital of Sun Yat-sen University, Guangzhou 510120, China; 2 Department of Clinical Immunology, The Third Affiliate Hospital of Sun Yat-sen University, Guangzhou 510630, China; 3 Guangdong Provincial Key Laboratory of Malignant Tumor Epigenetics and Gene Regulation, Sun Yat-sen Memorial Hospital of Sun Yat-sen University, Guangzhou 510120, China; 4 Department of Nephrology, Affiliated Hospital of Xuzhou Medical University, Xuzhou, Jiangsu 221000, China; 5 Department of Internal Medicine, Ohio State University College of Medicine, Columbus, OH 43210, USA; 6 Department of Medicine, Penn State College of Medicine, Hershey, PA 17033, USA

**Keywords:** CD39, CD8, T regulatory cell, lupus nephritis

## Abstract

Many patients with systemic lupus erythematosus (SLE) have lupus nephritis, one of the severe complications of SLE. We previously reported that CD8+CD103+ T regulatory cells induced *ex vivo* with transforming growth factor β (TGF-β) (iTregs) inhibited immune cells responses to ameliorate excessive autoimmune inflammation. However, the molecular mechanism(s) underlying the role of these CD8+ iTregs is still unclear. Here we identified that CD39, which is highly expressed on CD8+ iTregs, crucially contributes to the immunosuppressive role of the CD8+CD103+ iTregs. We showed that adoptive transfer of CD8+CD103+ iTregs significantly relieves the chronic graft-versus-host disease with lupus nephritis and CD39 inhibitor mostly abolished the functional activities of these CD8+ iTregs *in vitro* and *in vivo*. CD39+ cells sorted from CD8+CD103+ iTregs were more effective in treating lupus nephritis than CD39− partner cells *in vivo*. Furthermore, human CD8+ iTregs displayed increased CD103 and CD39 expressions, and CD39 was involved in the suppressive function of human CD8+ iTregs. Thus, our data implicated a crucial role of CD39 in CD8+CD103+ iTregs in treating lupus nephritis, and CD39 could be a new phenotypic biomarker for the identification of highly qualified CD8+ Tregs. This subpopulation may have therapeutic potential in patients with SLE nephritis and other autoimmune diseases.

## Teaser

This work demonstrates that CD39 plays a key role on CD8+CD103+ induced T regulatory cells in treating lupus nephritis. Using a chronic graft-versus-host disease lupus nephritis model and a series of *in vitro* experiments, Zhang et al. showed that CD39 is an excellent marker to identify the suppressive activity of CD8+CD103+ cells.

## Introduction

The pathogenesis underlying systemic lupus erythematosus (SLE) lies in the disturbance of immunities, consisting of abnormal numbers and functions of immune cells.^1^, ^2^, ^3^ Findings from several studies have identified reduced numbers and frequencies of T regulatory cells (Tregs) in SLE.^4^, ^5^, ^6^, ^7^ Additionally, Tregs correlated inversely with the disease activity of SLE.^8^ These Tregs are identified as a population of T cells able to control intense immune responses. However, because of the different gating strategies of Tregs, there were contradictory results on the frequencies of these cells.^9^, ^10^

The classic regulatory T cells are CD4+CD25+FOXP3+ T cells.^11^ Additionally, CD8+ Tregs are also able to inhibit the proliferation and effector function of effector lymphocytes,^12^ and they have drawn increased attention for treating autoimmune diseases.^13^, ^14^, ^15^ Interestingly, the subsets of CD8+ Tregs in each study are somewhat different. For example, CD8+CD122+ Tregs may correspond to CD4+CD25+ Tregs.^16^ Studies have found that CD8+CD122+ Tregs are fairly potent in immunosuppression.^17^, ^18^, ^19^ In addition, since CD28 is a major costimulatory receptor, CD8+CD28− T cells appear to negatively impact immune responses,^20^ and fewer CD8+ T cells expressed CD28 in SLE patients.^21^ Moreover, there were studies on other phenotypes of CD8+ Tregs. Differences in phenotypes are likely to be related to different disease models or methods to acquire Tregs. These CD8+ Tregs are usually reported to be decreased in SLE patients or favored for SLE therapy.^22^, ^23^ We previously reported that CD8+CD103+ Tregs generated *ex vivo* with TGF-β were notable for their potent suppressive capacity. Unlike CD4+Foxp3+ Tregs, these cells suppressed T cell responses regardless of Foxp3 expression, and they also played a role in the spontaneous liver tolerance and autoimmunosuppression of stimulatory graft-versus-host disease (GVHD) with a lupus-like syndrome.^24^, ^25^, ^26^, ^27^

CD103, the αEβ7 integrin, is a receptor for the epithelial cell-specific ligand E-cadherin. It has been reported to be associated with immune tolerance in transplantation, and it helps to distinguish the CD8+ Treg population from non-Tregs.^28^, ^29^ To determine if CD103 is a unique marker for CD8+ Tregs, we carried out an RNA sequencing (RNA-seq) analysis (NCBI SRA: PRJNA419054) to determine the differentially expressed genes between CD8+CD103− cells and CD8+CD103+ Tregs induced *ex vivo* with transforming growth factor β (iTregs). We noted that CD8+CD103+ iTregs have 3-fold more expression of Entpd1 than CD103− cells. Entpd1 is responsible for coding CD39, which has a major impact on the equilibrium of ATP and adenosine.^30^, ^31^ We therefore hypothesized that CD39 plays a functional role in CD8+CD103+ iTregs.

In this work, we observed that iTregs expressed a high level of CD39. The immunosuppressive capacity of CD8+CD103+ Tregs declined when a CD39 inhibitor was administered or the CD39 population was deleted. Moreover, the immunosuppressive effect of human CD8+ iTregs was also related to CD39 expression. Thus, CD39 provides a potential phenotypic marker to identify the induced CD8+ Tregs with high accuracy, and this has important clinical implications.

## Results

### CD8+ Tregs Induced *Ex Vivo* with TGF-β Exhibit a Potent Therapeutic Effect on Chronic GVHD Lupus Nephritis

**Figure 1:**
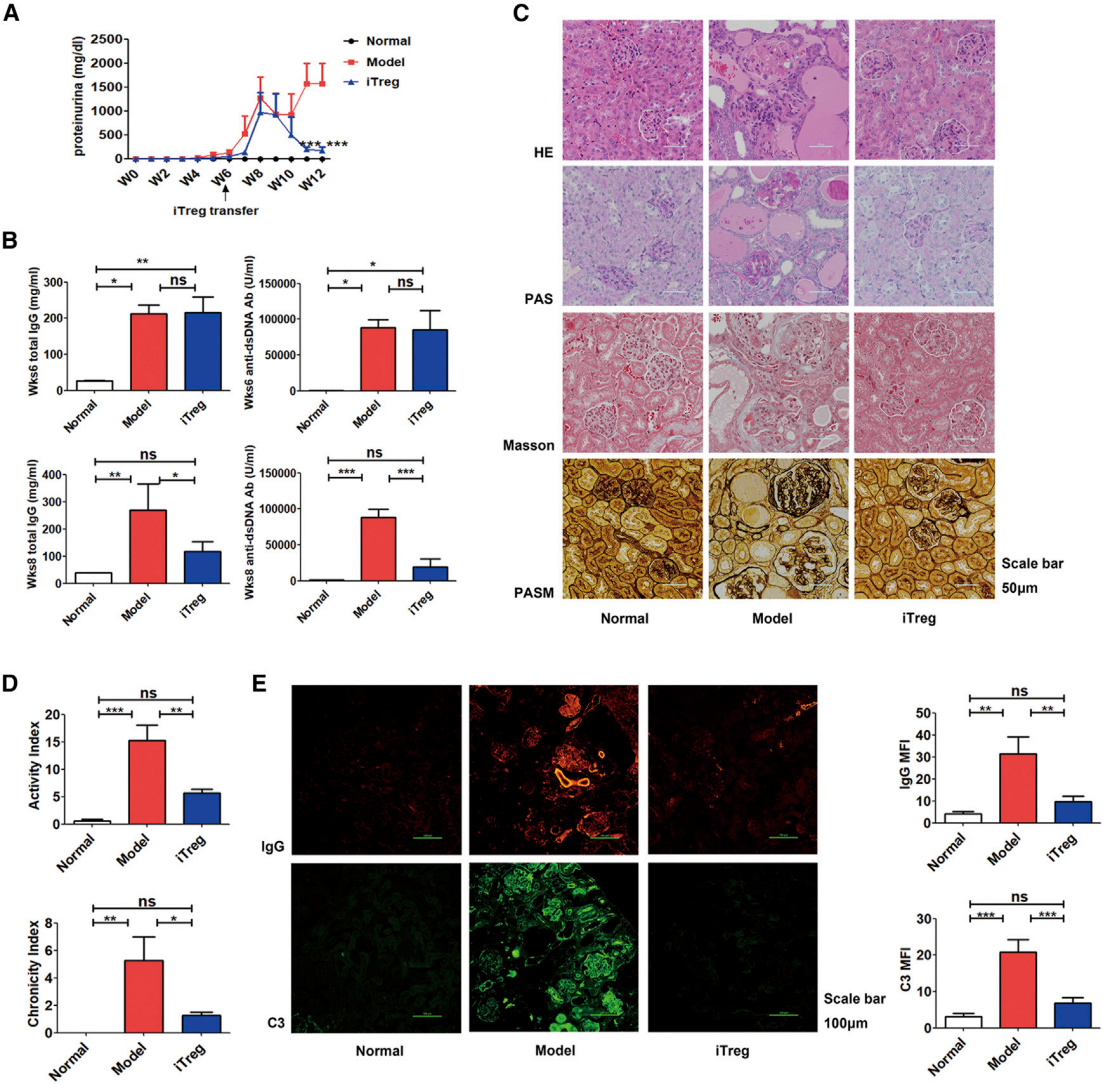
CD8+ Tregs Induced with TGF-β Exhibit a Potent Therapeutic Functionality on cGVHD Lupus CD8+ iTregs were adoptively transferred to cGVHD lupus nephritis mice at week 6. (A) CD8+ iTregs prevented the continuous rise in proteinuria in cGVHD lupus mice after 9 weeks. (B) The levels of anti-dsDNA antibody and total IgG in sera before treatment (at week 6, top) and after treatment (at week 8, bottom). (C and D) CD8+ iTregs alleviated the renal pathologic lesion (C) with lower disease activity and chronicity indices (D) at week 12. Scale bar: 50 μm. (E) CD8+ iTregs reduced IgG or C3 immune deposition in the glomeruli; IgG or C3 mean fluorescence intensity (MFI) was significantly lower in iTregs treatment group at week 12. Scale bar: 100 μm. The data indicate the mean ± SEM of four individuals (NS means no significance; *p < 0.05, **p < 0.01, ***p < 0.001).

To detect the immunosuppressive function of iTregs on lupus nephritis, we performed a chronic GVHD (cGVHD) lupus nephritis model induced in B6D2F1 mice by intravenously (i.v.) injecting DBA/2 spleen cells. In this study, we did show that mice had proteinuria at week 5 after DBA/2 cell transfer. Mice were selected to use as the lupus nephritis model when proteinuria occurred at >10 mg/dL. We infused iTregs into these model mice with defined proteinuria at 6 weeks after injecting DBA/2 spleen cells. iTreg infusion significantly ameliorated lupus nephritis. The levels of urine protein in mice in the iTreg treatment group began to decline at week 9, and levels were close to that of the normal group at week 12. As expected, proteinuria became gradually worse in the untreated model mice (Figure 1A). We also tested the levels of anti-double-stranded DNA (dsDNA) antibody and immunoglobulin G (IgG) in sera. We noted that the disease model mice had developed high levels of anti-dsDNA and IgG antibodies in sera before iTreg treatment, but their levels were significantly lower following iTreg treatment compared to the non-treatment control mice (Figure 1B).

For further evaluation of the glomerulonephritis, all mice were sacrificed at 12 weeks following the transfer of DBA/2 cells, and the kidney sections were examined histologically for the presence and severity of nephritis and for immune complex deposition by immunofluorescence. Compared with the model group, transfer of iTregs markedly ameliorated glomerulonephritis in cGVHD lupus mice (Figure 1C). Scores for both the activity index and chronicity index were markedly lower in the iTreg treatment group than those in the model group (Figure 1D). In addition, mice in the iTreg treatment group had significantly less IgG or C3 deposition in kidney than the mice in the model group. Moreover, mean fluorescence intensity (MFI) of the renal section was significantly lower in the CD8+CD103+ iTreg group versus the model group (Figure 1E).

### CD39 Is Highly Expressed on CD8+ Tregs Induced with TGF-β

The finding that CD8+ Tregs induced *ex vivo* with TGF-β have therapeutic functionality on cGVHD lupus nephritis stimulated us to further investigate the underlying molecular mechanisms responsible for the function of these iTregs. Besides increased expression of CD103, we also wondered whether there are other possible molecules that identify the functionality of the CD103+ Treg population. Thus, we carried out an RNA-seq analysis to determine the differentially expressed genes between CD103+ and CD103− cell populations.

**Figure 2:**
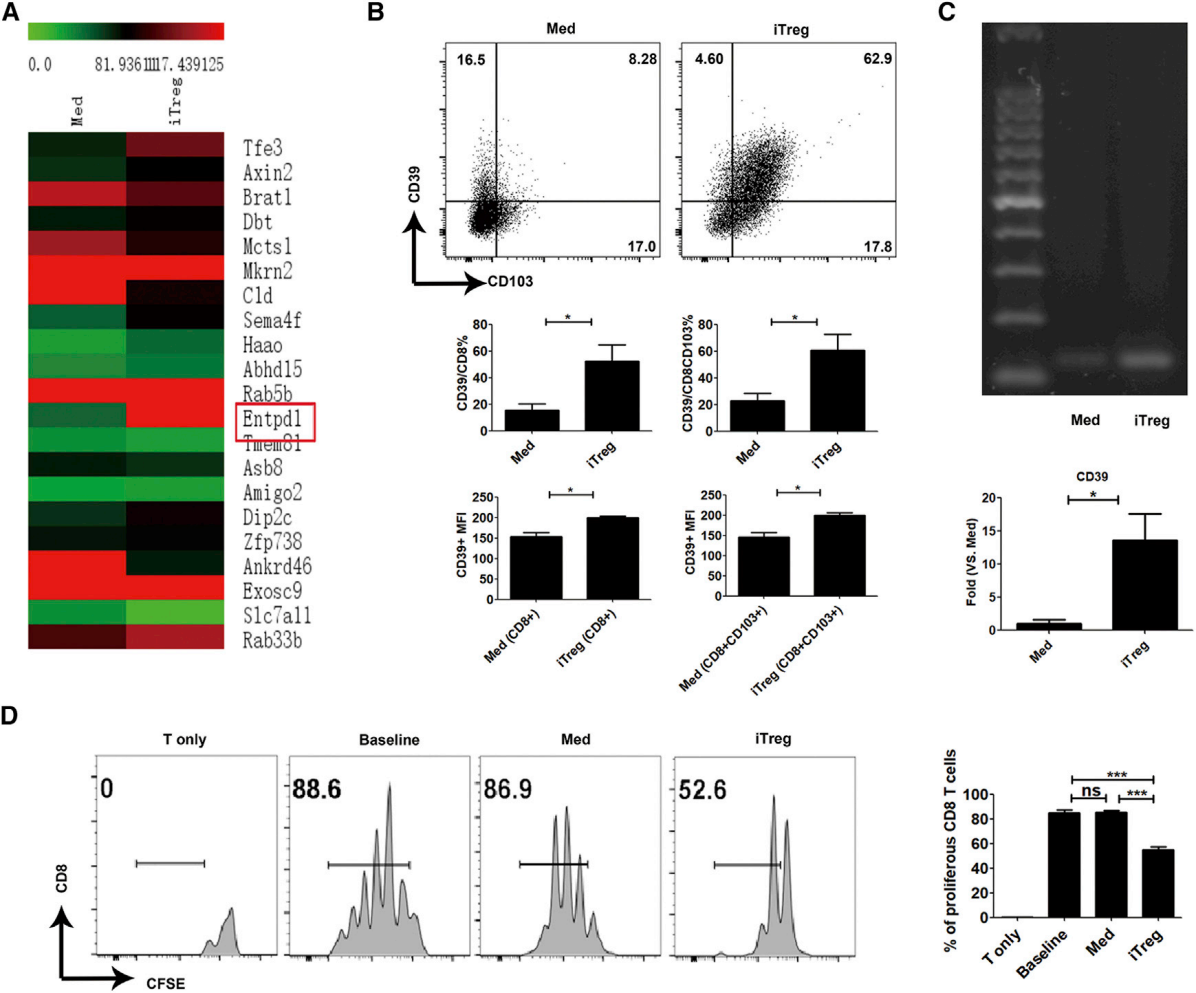
CD39 Is Highly Expressed on CD8+ Tregs Induced with TGF-β (A) CD8+CD103+ iTregs had significantly higher expression of Entpd1 compared with CD8+CD103− Med cells. (B) The expression level of CD39 was analyzed by flow cytometry. Med, medium cells; iTreg, induced T regulatory cells. CD39 expression is significantly greater on CD8+ iTregs than on CD8+ Med cells. (C) The mRNA level of CD39 in CD8+ iTregs was significantly higher than that on Med cells analyzed by PCR and qPCR. (D) CD8+ iTregs showed more potent suppression on T cell proliferation than Med cells *ex vivo*. The data are shown as the mean ± SEM of three independent experiments (NS means no significance; *p < 0.05, **p < 0.01, ***p < 0.001).

We noted that CD103+ iTregs have a 3-fold increase in *Entpd1* expression over CD103− cells, suggesting that CD39, which was coded by *Entpd1*, may be related to CD103+ iTreg function (Figure 2A). Using flow cytometry, we also validated that CD39 protein expressed on CD103+ iTregs was significantly higher than on CD103− cells (Figure 2B). In addition, we noted that the MFI of CD39 expression in iTregs was markedly higher than that in the control Med cells. The mRNA levels of CD39 were consistent with its protein levels (Figure 2C), implicating TGF-β in the induction of CD39 differentiation.

Additionally, *Igha*, *Tnfrsf11a*, and *Rps27a-ps2* were other top differentially expressed genes between these two Treg populations. As CD265 coded by *Tnfrsf11a* is involved in the nuclear factor κB (NF-κB)-signaling pathway,^32^ we also validated the expression of CD265 protein; unexpectedly, CD265 protein was undetectable in both CD103+ and CD103− iTreg populations (Figure S1). *Igha* codes immunoglobulin heavy constant α, while *Rps27a-ps2* is a pseudogene; we did not focus on these proteins since they have no connection to immunosuppression.^33^, ^34^ T cells that had been labeled with carboxyfluorescein succinimidyl ester (CFSE) enabled an analysis of quantitative cell proliferation.^35^ We found iTregs were more potent than Med cells to suppress the proliferation T responder cells (Figure 2D). We suggest that the difference in CD39 expression may contribute to the different functional characteristics between CD8+ iTregs and CD8+ Med cells.

### CD39 Expression in CD8+ iTregs Is Essential for the Suppressive Function on Proliferation and Differentiation of T Cells *Ex Vivo*

**Figure 3:**
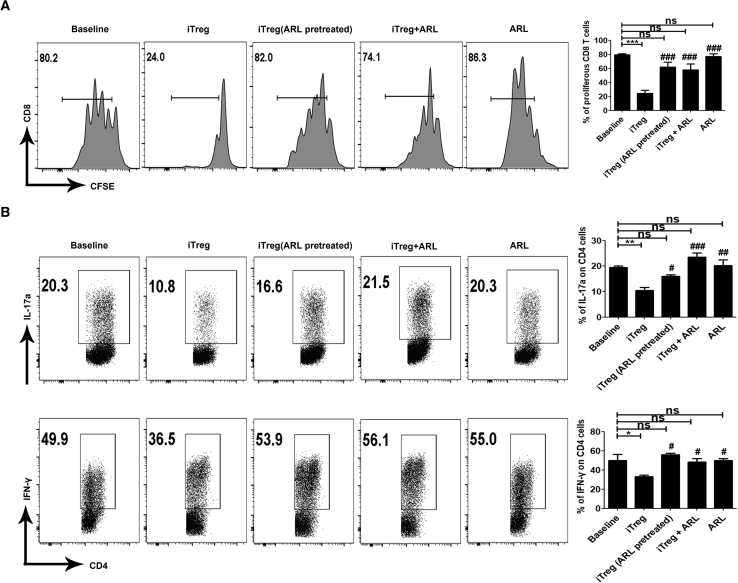
CD39 in CD8+ iTregs Is Essential for the Suppressive Function on Proliferation and Differentiation of T Cells *Ex Vivo* CD39 inhibitor ARL 67156 (ARL) was pretreated with CD8+ iTregs or added into the cell culture system in a suppression assay. (A) ARL weakened the suppressive function of iTregs on CD4+ T cell proliferation. (B) ARL eliminated the suppressive function of iTregs on the secretion of IL-17a or IFN-γ from CD4+ cells. The data are shown as the mean ± SEM of three independent experiments (NS means no significance; *p < 0.05, **p < 0.01, ***p < 0.001, each group versus baseline; ^#^p < 0.05, ^##^p < 0.01, ^###^p < 0.001, each group versus CD8+ iTregs).

We then directly explored the role of CD39 expressed on CD8+ iTregs in suppressing T cell responses. ARL 67156 (ARL), a CD39 inhibitor, was first used to eliminate the effect of CD39 in CD8+ iTregs. As expected, blockade of CD39 in CD8+ iTregs markedly abolished the suppressive effect of CD8+ iTregs on CD8+ T responder cell proliferation in a co-culture system. In addition, iTregs pretreated with ARL were also diminished in their suppressive function. As ARL was washed off after pretreatment, this experiment helps to exclude the possibility that ARL affects T responder cell proliferation (Figure 3A). To further evaluate the effect of CD39 expression of CD8+ iTregs on T cell response, we conducted Th17 and Th1 differentiation assays. We found that iTregs inhibit both interleukin (IL)-17a and interferon (IFN)-γ secretion from CD4+ cells but their suppressive function is impaired after ARL pretreatment (Figure 3B).

To exclude the possibility that the cell culture may be differently stimulated by ARL, we added ARL into cell cultures without iTregs. The addition of ARL changed neither the proliferation nor the differentiation of T cells *ex vivo*. These data suggest that CD39 in CD8+ iTregs seems essential for the suppressive function on the proliferation and differentiation of T cells *ex vivo*.

### CD39 Expression in CD8+ iTregs Plays an Important Role in Inducing Immune Tolerance for cGVHD Lupus

Due to the important role of CD39 expression in CD8+ iTregs *in vitro*, we next asked whether CD39 expression is crucial for CD8+ iTregs in treating lupus nephritis *in vivo*. CD8+CD103+CD39+ T cells (CD39+) and CD8+CD103+CD39− T cells (CD39−) were acquired from CD8+ iTregs through cell sorting by a FACSAria III. These cells were adoptively transferred into the lupus mice at 6 weeks after the induction of lupus-like syndromes through injecting DBA/2 spleen cells. In another group, we also treated mice with CD8+ iTregs pretreated with ARL.

**Figure 4:**
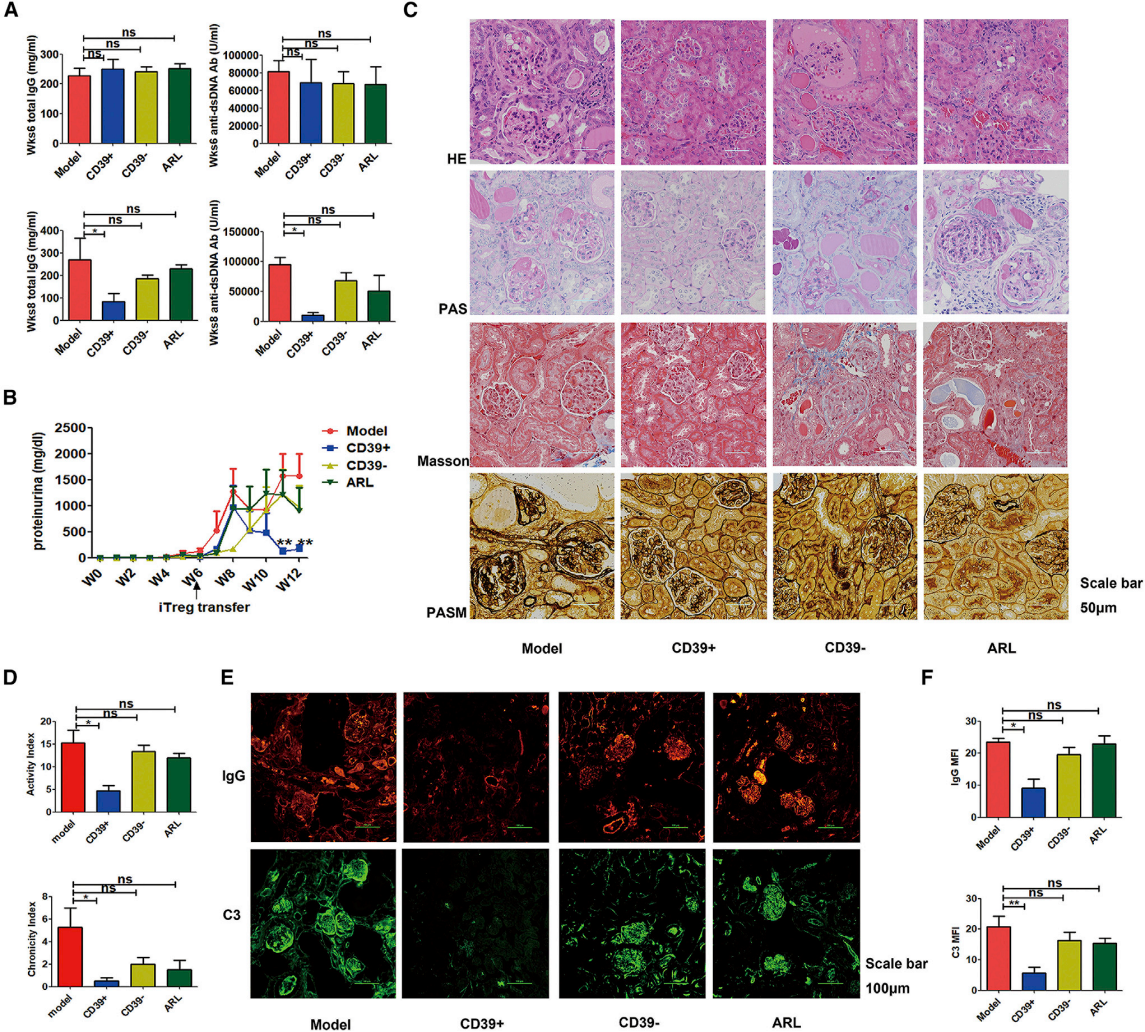
CD8+CD103+CD39+ iTregs Had a More Potent Therapeutic Effect on Lupus Nephritis Mice Than CD8+CD103+CD39− T Cells or CD8+ iTregs Pretreated with ARL CD8+CD103+CD39+ T cells (CD39+) and CD8+CD103+CD39− T cells (CD39−) were acquired by sorting from iTregs. CD8+CD103+CD39+ T cells, CD8+CD103+CD39− T cells, or CD8+ iTregs pretreated with ARL (ARL) were adoptively transferred to cGVHD lupus nephritis mice at week 6. (A) The levels of anti-dsDNA antibody and total IgG in sera before treatment (at week 6, top) and after treatment (at week 8, bottom). (B) CD8+CD103+CD39− T cells or CD8+ iTregs pretreated with ARL failed to prevent the continuous rise in proteinuria in cGVHD lupus mice after 9 weeks. (C and D) CD8+CD103+CD39+ T cells alleviated the renal pathologic lesion (C), with lower disease activity and chronicity indices (D) than CD8+CD103+CD39− T cells and CD8+ iTregs pretreated with ARL at week 12. Scale bar: 50 μm. (E) Mice in the CD39+ group had less IgG and C3 immune deposition in the glomeruli than mice in the CD39− group and the ARL group at week 12. Scale bar: 100 μm. (F) IgG and C3 (MFIs) were significantly lower in the CD39+ group than those in the CD39− group and the ARL group at week 12. The data show the mean ± SEM of four individuals (NS means no significance; *p < 0.05, **p < 0.01).

Examination of sera in mice 6 weeks following the adoptive transfer of DBA/2 cells revealed that IgG and anti-dsDNA antibody levels in the CD39+ cell treatment group were comparable to those in other groups, but they were markedly decreased after cell treatment. By contrast, mice that received CD39− or ARL treatment cells still maintained high levels of IgG and anti-dsDNA antibody, similar to model mice at 8 weeks (Figure 4A). As expected, proteinuria was gradually increased after DBA/2 cell transfer in the model group. Treatment of CD39− T cells had no influence on proteinuria. Conversely, proteinuria was gradually reduced at 10 weeks after DBA/2 cell transfer when lupus mice had received CD39+ treatment. Interestingly, the effect of CD8+CD103+ iTreg on proteinuria decreased when these cells were pretreated with the CD39 inhibitor (Figure 4B).

We further used histological analysis to determine nephritis severity 12 weeks after DBA/2 cell transfer. These methods help to analyze glomerular cell proliferation, crescent formation, glomerular sclerosis, and interstitial fibrosis in the lupus model. CD8+CD39+ iTregs, but not control cell, treatment also significantly decreased the severity of lupus nephritis (Figure 4C), and it resulted in a significantly lower degree of disease activity and chronicity (Figure 4D). Immunofluorescence analysis showed massive pathological IgG and C3 deposits in the glomeruli in the mice of CD39− and ARL groups that were similar to the lupus group, while markedly decreased deposition was seen in the CD39+ treatment mice (Figure 4E). The MFI of IgG or C3 in the CD39+ treatment group was also significantly lower than that in the other 3 groups (Figure 4F). These results show that CD39 expression in CD8+ iTregs plays an important role in inducing immune tolerance for lupus nephritis.

### CD39 Expression Is Related to the Suppressive Functionality of CD8+ iTregs from Human PBMCs

**Figure 5:**
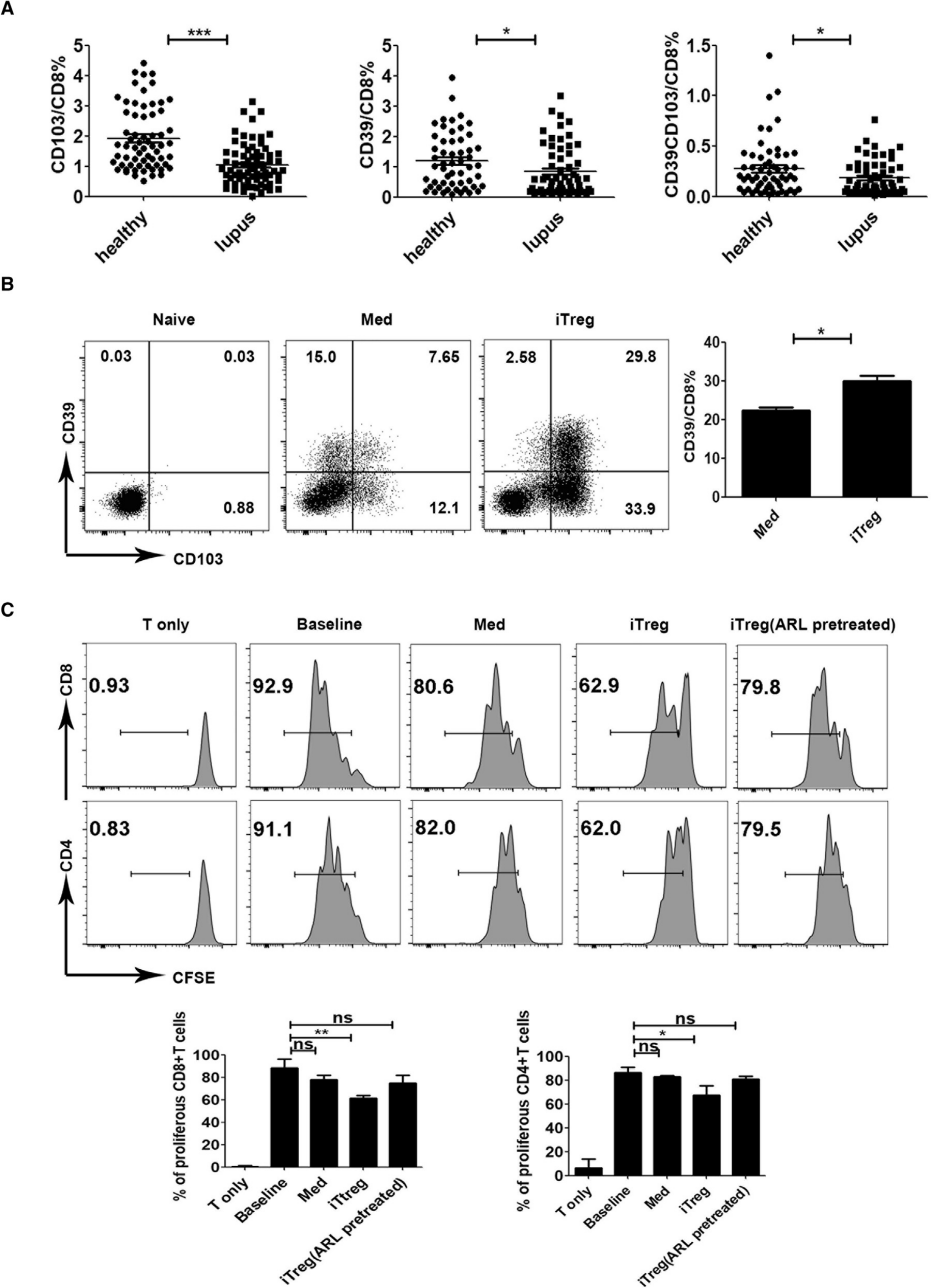
CD39 Expression Is Related to the Suppressive Functionality of CD8+ iTregs from Human PBMCs (A) Percentages of CD8+ T cells expressing the CD103 or CD39 in lupus patients were lower than those of healthy control subjects. (B) CD103 and CD39 expressions were increased after TGF-β stimulating on CD8+ naive T cells from PBMCs of healthy humans. The data show the mean ± SEM of three independent experiments. (C) CD8+ iTregs showed more potent suppression of T cell proliferation than Med cells and CD8+ iTregs pretreated with ARL *ex vivo*. The data indicate the mean ± SEM of three independent experiments (NS means no significance; *p < 0.05, **p < 0.01, ***p < 0.001).

To determine the clinical relevance of this finding, we extended the study from mouse cells to human cells. Samples of peripheral blood were obtained by venipuncture from lupus patients and healthy donors. We analyzed the expressions of CD103 and CD39 in peripheral blood mononuclear cells (PBMCs) by flow cytometry. Percentages of CD8+ T cells expressing the CD103 or CD39 in lupus patients were significantly decreased when compared with those of healthy controls (HCs). In addition, a lower proportion of the CD8+CD103+CD39+ T cell subpopulation was observed in lupus patients versus that in HCs (Figure 5A). These results suggest some connections between lupus development and the decline in the percentage of CD8+CD103+CD39+ T cells.

We wondered whether CD8+ naive T cells from humans possessed the same ability to be induced into Tregs with TGF-β as in mice. We sorted CD8+ naive T cells (CD8+CD45RA+CCR7+ cells) by a FACSAria III from healthy donor PBMCs. Then CD8+ naive T cells were stimulated with (iTregs) or without TGF-β (Med cells). We evaluated the surface expression of CD103 and CD39 in cells before and after stimulating for 5 days. CD8+ naive cells hardly expressed CD103 and CD39, while CD103 and CD39 were markedly increased in iTregs relative to Med cells (Figure 5B). We then harvested Med cells and iTregs to assay their suppressive activities. iTregs exhibited a suppressive effect on the proliferation of T cells from human PBMCs *in vitro*, but Med cells did not (Figure 5C). Likewise, ARL also impaired the suppressive function of iTregs, suggesting that CD39 expression is a crucial feature for conferring suppressive activity on CD8+ iTregs from human PBMCs.

## Discussion

Treg-mediated suppression serves as a vital mechanism for the negative regulation of immune-mediated inflammation, and it features prominently in autoimmune and inflammatory disorders.^36^, ^37^, ^38^ Several lines of experimentation have provided the proof that a lack of Tregs is the cause of fatal autoimmunity.^39^, ^40^, ^41^, ^42^ CD8+ Tregs, one subset of Tregs, have been reported to display potent therapeutic effect in some diseases, such as lupus and GVHD.^43^, ^44^, ^45^ We have previously reported that CD8+CD103+ Tregs induced *ex vivo* with TGF-β suppress the T cell response regardless of Foxp3 expression.^24^ As CD103 has other roles like cell migration, it is necessary to further identify the molecular marker to distinguish the Treg subset from the CD103+ population.

RNA-seq analysis helps to identify some important information on a molecular basis of immunosuppression between CD8+CD103+ and CD8+CD103− cell populations.^27^ *Entpd1*, *Igha*, *Itgae*, *Tnfrsf11a*, and *Rps27a-ps2* were the top differentially expressed genes between these two Treg populations. We focused on two genes that code immunoregulatory proteins. Validation experiments demonstrated that only CD39 protein is highly expressed in CD103+ iTregs. Our *in vitro* and *in vivo* experiments further confirmed that the CD39+ iTreg population is a potent suppressor to inhibit inflammation and lupus nephritis. CD265, known as a receptor activator of NF-κB, is coded by gene *Tnfrsf11a*.^32^ *Tnfrsf11a* is highly expressed but CD265 protein is undetectable in CD8+CD103+ iTregs. Interestingly, Liu et al.^46^ found that NF-κB in Murphy Roths lupus/lymphoproliferation (MRL/lpr) mouse glomerular endothelial cells activated by lipopolysaccharide (LPS) was suppressed by CD8+ iTregs so that glomerular endothelial cells could be prevented from injury. The exact role of CD265 in CD8+CD103+ Tregs remains to be further documented in the future. Moreover, there may be other molecule(s) responsible for the immunosuppressive function mediated by CD8+CD103+ Tregs.

Indeed, we observed that CD103+ iTregs had a 3-fold increase in Entpd1 expression relative to CD103− cells, and CD39 protein coded by Entpd1 gene was also significantly upregulated on CD8+CD103+ Tregs in our flow cytometry study. Moreover, we and others have previously reported that CD39 regulates the immunosuppressive function of mesenchymal stem cells.^30^, ^47^, ^48^, ^49^ It is reasonable to make a hypothesis that CD39 may be a specific marker for CD8+CD103+ iTregs, which may also play an important role in the immunosuppressive function of CD8+CD103+ iTregs. CD39 and CD73 degrade extracellular ATP to adenosine together. Since ATP and adenosine have opposing effects on inflammation, the local expression of CD39 and CD73 can shape the quality of immune responses.^50^ Regateiro et al.^51^ reported that TGF-β induced the expressions of both CD39 and CD73 in T cells. However, we found that CD73 expression was not different among CD8+ naive T cells, CD8+ Med cells, and CD8+CD103+ iTregs (Figure S2); thus, CD39, but not CD73, identifies the CD8+ iTreg population that displays advantages in suppressing immune responses and responding to cell therapy in lupus nephritis.

Lupus patients exhibit increased levels of ATP, which results in activation of the inflammasome and the consequent release of cytokines associated with disease pathogenesis.^52^, ^53^ To avoid ATP-induced pathological effects, CD39 catalyzes the phosphohydrolysis of ATP into adenosine, which is a potent immune regulator of cells that express A2 and A3 receptors, such as lymphocytes.^54^ In addition, Knight et al.^55^ reported that CD39-knockout mice develop more severe lupus, demonstrating that ectonucleotidase mediates the suppression of lupus autoimmunity. Some studies showed that adenosine receptor activation arrests glomerulonephritis inflammation in lupus mice or other immune-associated chronic inflammation models.^56^, ^57^ Using innovative technologies, we now have shown that CD39 plays an important role in CD8+CD103+ iTregs to exert their immunosuppression. It is likely that Tregs mediating the elimination of ATP and generation of adenosine, dependent on CD39, are important mechanisms for inhibiting lupus nephritis. Moreover, CD39 can be a biomarker for the identification of CD8+ Tregs. Since CD8+CD103+CD39− cells have less or no therapeutic effect, they should be excluded to optimize the clinical application of Treg therapy for lupus. Using human cells further supports this possibility.

Although the functional activity of CD8+CD103+CD39+ was mainly documented in the animal models in this study, our finding could have an important clinical implication on human SLE. Previous studies have demonstrated that active TGF-β production is markedly decreased in lupus patients.^58^, ^59^, ^60^ The decrease or lack of active TGF-β could contribute to the reduction in CD8+CD103+CD39+ Tregs that promotes SLE development. In addition, a previous study found that CD8+CD103+ iTregs have potent suppressive activity both *in vitro* and *in vivo*;^24^ the functional activity of this suppressor cell population may be also related to CD39 expression.

Taken together, our data suggest that CD39 expression is responsible for CD8 Tregs induced with TGF-β to inhibit lupus nephritis in cGVHD and CD39 is a novel biomarker for the identification of CD8+ Tregs. Manipulation of CD8+CD103+CD39+ may have a therapeutic promise in treating patients with SLE with nephritis and other autoimmune diseases.

## Materials and Methods

### Mice and Human Subjects

The 6- to 8-week-old female C57BL/6 (B6) mice were purchased from Guangdong Medical Laboratory Animal Center (Guangzhou, China), and the 6-week-old female DBA/2 mice and 6-week-old female (C57BL/6 × DBA/2) B6D2DF1 mice were purchased from Vital River (Beijing, China). This study was carried out in accordance with the recommendations of Sun Yat-sen University for the Use and Care of Animals (approval SYSU-IACUC-2018-000046). The protocol on human study was approved by the Ethics Committee of Sun Yat-sen Memorial Hospital and conducted in accordance with the principles enshrined in the Declaration of Helsinki (2013). Written informed consent was provided by all subjects.

### Flow Cytometry

The following fluorescent antibodies (Abs) from BioLegend (San Diego, CA, USA) were used for fluorescent cytometry analysis. Cell subsets were stained with monoclonal Abs (mAbs) and isotype control and analyzed on a BD LSRFortossa flow cytometer (BD Biosciences, San Diego, CA, USA). For intracellular staining, cells were stained with surface antigen and further fixed and permeabilized for intracellular staining. For inflammatory cytokine staining, cells were prepared and cultured with Phorbol 12-Myristate 13-Acetate (50 ng/mL; Sigma-Aldrich, Taufkirchen, Germany) and ionomycin (500 ng/mL; Sigma-Aldrich) for 5 h in the presence of brefeldin A (5 μg/mL; BioLegend) for the last 4 h. Cytokine expression was measured by fluorescence-activated cell sorting (FACS).

### The Generation of CD8+ Med Cells, CD8+ Tregs, CD8+CD103+CD39− Cells, and CD8+CD103+CD39+ Cells

CD8+ Med cells and CD8+ Tregs were generated with (CD8+ iTreg) or without (CD8+ Med) recombinant human TGF-β (rhTGF-β) (2 ng/mL; R&D Systems, San Diego, CA, USA), as described in our previous study.^24^ They were harvested and sorted for selection of different subsets using a FACSAria III (BD Biosciences) high-speed cell sorter. In some cultures, CD39 inhibitor ARL 67156 (50 μM; Sigma-Aldrich) was added.

### *In Vitro* Suppression Assay

Fresh T responder cells labeled with CFSE (BioLegend) were co-cultured with non-T cells, which were incubated with mitomycin C (50 μg/mL; Sigma-Aldrich) for 20 min at 37°C, followed by washing with complete RPMI. T responder cells were stimulated with anti-CD3 mAb for 3 days with or without conditioned cells. T cell proliferation was determined by the CFSE dilution rate.

### cGVHD Lupus Nephritis Model and Adoptive Transfer

A cGVHD lupus nephritis model was induced in B6D2F1 mice, as described previously, by injecting 80 × 10^6^ DBA/2 spleen cells.^27^, ^61^ Proteinuria was determined with Semiquantitative Albustix paper (Gaoerbao, Guangzhou, China). The level of IgG and dsDNA serum was determined by ELISA. The mice that had proteinuria were selected for use as the lupus nephritis model. In week 6, 3 × 10^6^ different cells in PBS were transferred into cGVHD lupus mice, respectively. Normal and model group mice received the same volume of PBS. There were 4 mice for each group in one experiment, and two additional experiments were repeated with similar results.

### Pathology and Immunofluorescence

The kidney tissues of mice were processed for light and immunofluorescent microscopy. The light-microscopic slides were stained with H&E, Masson, periodic acid-Schiff stain (PAS), or periodic acid-methenamine stain (PASM), and they were used to calculate the activity and chronicity indices of different groups.^62^, ^63^ Immunofluorescence slides were stained with IgG (Abcam, Cambs, UK) or C3 (Abcam), observed with a fluorescence microscope (Nikon, Tokyo, Japan), and the MFIs of glomeruli in different groups were calculated using ImageJ sofware (NIH, USA).

### Real-Time PCR

Samples were run in triplicate. Primer sequences were as follows: CD39, 5′-AGT TAG AGG AAT GCC AAG TGA A-3′ and 5′-GTG ATG CTT GGA TGT TGG TAT C-3′; and CD265, 5′-CTG AAA AGC ACC TGA CAA AAG A-3′ and 5′-CTG TGT AGC CAT CTG TTG AGT T-3′.

### Western Blotting

Cells were washed twice with PBS and lysed on ice in radioimmunoprecipitation assay (RIPA) buffer (Cwbio, Beijing, China) and protease inhibitors (Roche, Basel, Switzerland). The cells were then centrifuged, and the collected supernatants were boiled and electrophoresed on a 10% SDS polyacrylamide gel. About 30 μg total protein from a cell lysate was loaded for data detection. Proteins were electro-transferred to membranes and incubated overnight at 4°C with anti-CD265 (Abcam) or glyceraldehyde-3-phosphate dehydrogenase (GAPDH; Abcam). Subsequently, the membranes were incubated at room temperature for 1 h with the anti-mouse IgG (Boster, Wuhan, China) or anti-rabbit IgG (Boster). Bands were detected using the Supersignal west Pico Plus chemiluminescent substrate (Thermo Scientific, Rockford, IL, USA). Western blot results were normalized to the expression of GAPDH and analyzed using the ImageJ software.

### Statistical Analysis

Data were expressed as mean ± SEM unless otherwise indicated. Data were analyzed using the unpaired t test for comparison between two groups or ANOVA for comparison among multiple groups as appropriate in GraphPad Prism 5. Comparison between two groups in multiple groups used the Bonferroni correction. Differences were considered statistically significant when p < 0.05.

## Author Contributions

A.X. and S.G.Z. designed the research topic and charge correspondence. X.Z. and S.G.Z. wrote the manuscript. X.Z., X.O., and Z.X. designed and carried out all the experiments. J.C., Q.H., T.X., and Y.L. helped carry out the experiments. J.W. provided assistance and guidance on the experiments. N.O. provided manuscript modification.

## Conflicts of Interest

The authors declare no competing interests.

## Supplemental Information

Document S1. Figures S1 and S2

Document S2. Article plus Supplemental Information
