# Supplementary material for: CD8+CD103+ iTregs Inhibit Chronic Graft-versus-Host Disease with Lupus Nephritis by the Increased Expression of CD39
Source: Mol Ther. 2019 Jul 26;27(11):1963–73. doi: 10.1016/j.ymthe.2019.07.014 (PMC6838901; doi:10.1016/j.ymthe.2019.07.014)
Supplement: Document S1. Figures S1 and S2 [file mmc1.pdf]

YMTHE, Volume *27*

Supplemental Information

CD8+CD103+ iTregs Inhibit Chronic Graft-versus-Host Disease with Lupus Nephritis by the Increased Expression of CD39

Xiao Zhang, Xia Ouyang, Zhenjian Xu, Junzhe Chen, Qiuyan Huang, Ya Liu, Tongtong Xu, Julie Wang, Nancy Olsen, Anping Xu, and Song Guo Zheng

Figure S1

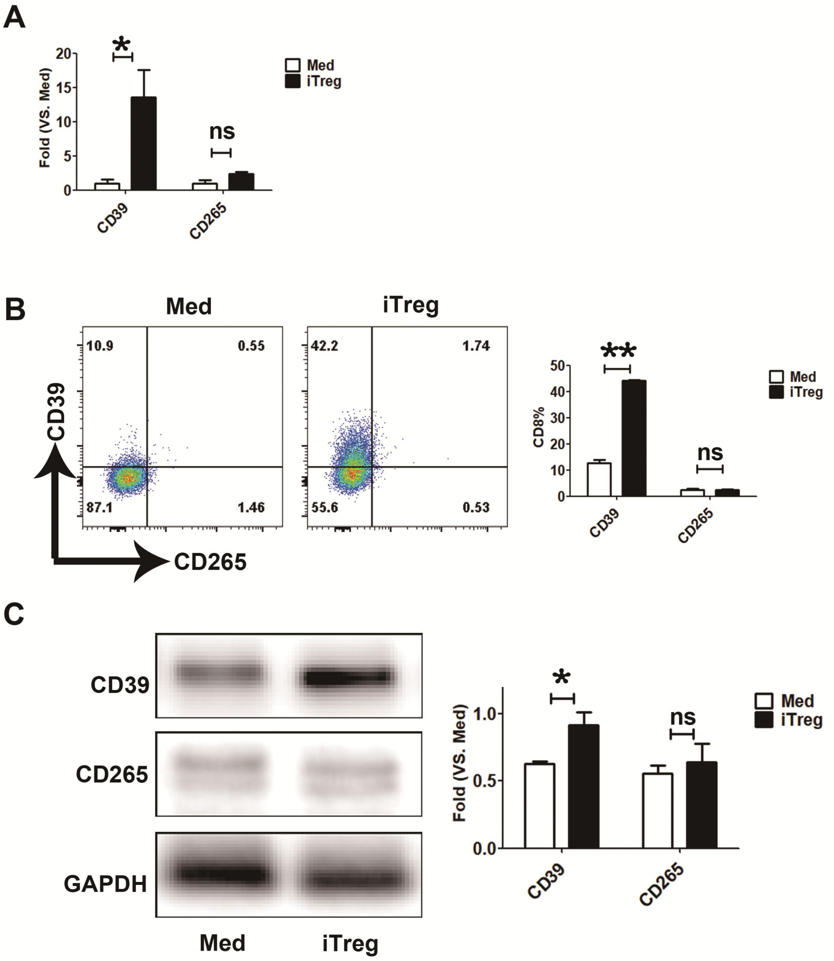

Figure S1 | CD265 protein was undetectable in both CD103+ and CD103- iTreg populations. CD39 and CD265 expression on Med cells and CD8+CD103+ iTregs were detected by real-time PCR (A), flow cytometry (B) and western blotting (C). The data indicate the mean ± SEM of three independent experiments. (NS means no significance, **P*<0.05, ***P*<0.01)

Figure S2

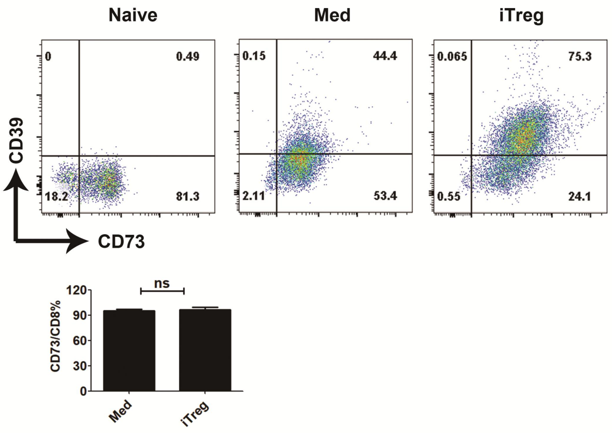

Figure S2 | CD73 expression was not different among CD8+ naive T cells, CD8+ Med cells and CD8+CD103+ iTregs. CD8+ naive cells were isolated from C57BL/6, stimulated with ($CD8^+CD103^+$ iTreg) or without TGF-β ($CD8^+CD103^-$ med) for 72 hours. The percentage of CD73+ cells in CD8+ cells was detected by flow cytometry. The data indicate the mean ± SEM of three independent experiments. (NS means no significance)
